# Targeting BCR-ABL-Independent TKI Resistance in Chronic Myeloid Leukemia by mTOR and Autophagy Inhibition

**DOI:** 10.1093/jnci/djx236

**Published:** 2017-11-20

**Authors:** Rebecca Mitchell, Lisa E M Hopcroft, Pablo Baquero, Elaine K Allan, Kay Hewit, Daniel James, Graham Hamilton, Arunima Mukhopadhyay, Jim O’Prey, Alan Hair, Junia V Melo, Edmond Chan, Kevin M Ryan, Véronique Maguer-Satta, Brian J Druker, Richard E Clark, Subir Mitra, Pawel Herzyk, Franck E Nicolini, Paolo Salomoni, Emma Shanks, Bruno Calabretta, Tessa L Holyoake, G Vignir Helgason

**Affiliations:** 1 Glasgow Polyomics, Institute of Cancer Sciences, University of Glasgow, Glasgow, UK; 2 Wolfson Wohl Cancer Research Centre, Institute of Cancer Sciences, University of Glasgow, Glasgow, UK; 3 Paul O’Gorman Leukaemia Research Centre, Institute of Cancer Sciences, University of Glasgow, Glasgow, UK; 4 Scottish National Blood Transfusion Service, Gartnavel General Hospital, Glasgow, UK; 5 Cancer Research UK, Beatson Institute, Garscube Estate, Glasgow, UK; 6 Faculty of Health and Medical Sciences, University of Adelaide, Adelaide, Australia and Imperial College, London, UK; 7 Strathclyde Institute of Pharmacy and Biomedical Sciences, University of Strathclyde, Glasgow, UK; 8 Hématologie Clinique 1G, Centre Hospitalier Lyon Sud, Pierre Bénite, France; 9 Division of Hematology and Medical Oncology, Oregon Health and Science University, Knight Cancer Institute, Portland, OR; 10 Institute of Translational Medicine, Department of Molecular and Clinical Cancer Medicine, University of Liverpool, UK; 11 Department of Haematology, Milton Keynes Hospital NHS Foundation Trust, Milton Keynes, UK; 12 Institute of Molecular, Cell and Systems Biology, College of Medical, Veterinary and Life Sciences, University of Glasgow, UK; 13 Samantha Dickson Brain Cancer Unit, UCL Cancer Institute, Paul O'Gorman Building, London, UK; 14 Department of Cancer Biology, Kimmel Cancer Center, Thomas Jefferson University, Philadelphia, PA

## Abstract

**Background:**

Imatinib and second-generation tyrosine kinase inhibitors (TKIs) nilotinib and dasatinib have statistically significantly improved the life expectancy of chronic myeloid leukemia (CML) patients; however, resistance to TKIs remains a major clinical challenge. Although ponatinib, a third-generation TKI, improves outcomes for patients with BCR-ABL-dependent mechanisms of resistance, including the T315I mutation, a proportion of patients may have or develop BCR-ABL-independent resistance and fail ponatinib treatment. By modeling ponatinib resistance and testing samples from these CML patients, it is hoped that an alternative drug target can be identified and inhibited with a novel compound.

**Methods:**

Two CML cell lines with acquired BCR-ABL-independent resistance were generated following culture in ponatinib. RNA sequencing and gene ontology (GO) enrichment were used to detect aberrant transcriptional response in ponatinib-resistant cells. A validated oncogene drug library was used to identify US Food and Drug Administration–approved drugs with activity against TKI-resistant cells. Validation was performed using bone marrow (BM)–derived cells from TKI-resistant patients (n = 4) and a human xenograft mouse model (n = 4–6 mice per group). All statistical tests were two-sided.

**Results:**

We show that ponatinib-resistant CML cells can acquire BCR-ABL-independent resistance mediated through alternative activation of mTOR. Following transcriptomic analysis and drug screening, we highlight mTOR inhibition as an alternative therapeutic approach in TKI-resistant CML cells. Additionally, we show that catalytic mTOR inhibitors induce autophagy and demonstrate that genetic or pharmacological inhibition of autophagy sensitizes ponatinib-resistant CML cells to death induced by mTOR inhibition in vitro (% number of colonies of control[SD], NVP-BEZ235 vs NVP-BEZ235+HCQ: 45.0[17.9]% vs 24.0[8.4]%, *P* = .002) and in vivo (median survival of NVP-BEZ235- vs NVP-BEZ235+HCQ-treated mice: 38.5 days vs 47.0 days, *P* = .04).

**Conclusion:**

Combined mTOR and autophagy inhibition may provide an attractive approach to target BCR-ABL-independent mechanism of resistance.

Chronic myeloid leukemia (CML) is caused by a reciprocal translocation giving rise to the Philadelphia (Ph) chromosome within a hemopoietic stem cell (1). This leads to transcription/translation of BCR-ABL, a constitutively active tyrosine kinase (2). CML usually presents in a chronic phase (CP), before progressing to accelerated phase (AP) and terminal blast crisis (BC) if left untreated. Imatinib has statistically significantly improved life expectancy by inducing cytogenetic and molecular responses in the majority of patients in CP (3). However, the pathway to “cure” has been tempered by drug intolerance, insensitivity of CML stem cells to TKIs (4–7), and drug resistance (8,9).

The mechanisms of drug resistance have been extensively investigated and can be classified as BCR-ABL dependent or independent. It is known that approximately 50% of patients who relapse on imatinib have mutations within the ABL kinase domain, affecting imatinib binding within the kinase pocket (10). Dasatinib, nilotinib, and/or bosutinib have activity against the majority of imatinib-resistant mutants, except T315I (11). Although the development of a TKI active against the T315I mutant has proven challenging, ponatinib (AP24534), a third-generation TKI, has activity against T315I in vitro (12) and in patients (13,14). Ponatinib was tested in the PACE clinical trial in patients with the T315I mutation or who are resistant/intolerant to either dasatinib or nilotinib. Findings from PACE show that major molecular response (MMR) is achieved in 56% of CP patients with the T315I mutation (14), although a proportion of patients will ultimately develop or be proven to have ponatinib-resistant disease.

Patients whose disease fails multiple TKI treatments without having ABL kinase domain mutations predominantly represent a population with BCR-ABL-independent mechanisms of resistance. For this group of patients, the treatment options are very limited, and only 27% of “resistant/intolerant” patients achieved MMR in the PACE trial (14). Although much less is known about BCR-ABL-independent resistance, a recent genetic study has shown that it can vary between individuals, often suggesting re-activation of signaling pathways involved in CML pathogenesis (15). Additionally, studies have shown that increased FGF2 in the BM (16) or activation of LYN (17,18) may be responsible for the survival of cells following BCR-ABL inhibition. However, ponatinib, which has activity against FGF receptor and LYN kinase (12), has been shown to overcome FGF2-mediated resistance in CML patients without kinase domain mutations (16) and to be effective against many imatinib-resistant CML cell lines (19), highlighting the importance of using ponatinib as the TKI of choice for investigation of acquired BCR-ABL-independent resistance in CML.

The goals of the current study were to examine what drives BCR-ABL-independent resistance and identify clinically relevant oncology compounds with activity against ponatinib-resistant cells.

## Methods

### Transplantation Experiments

Human KCL22^Pon-Res^ cells, labeled with lentiviral firefly luciferase, were transplanted via tail vein injection into eight- to 12-week-old female NSG mice (four to six mice were assigned per drug arm per experiment). For in vivo treatment, after one week, the mice were treated with vehicle control, HCQ, NVP-BEZ235, or the combination of NVP-BEZ235/HCQ for four to five weeks.

### Ethics Statements

CML and normal samples (n = 4 and n = 5, respectively) required informed consent in accordance with the Declaration of Helsinki and approval of the National Health Service (NHS) Greater Glasgow Institutional Review Board. Ethical approval has been given to the research tissue bank (REC 15/WS/0077) and for using surplus human tissue in research (REC 10/S0704/60). Animal work was carried out with ethical approval from the University of Glasgow under the Animal (Scientific Procedures) Act 1986. Animal experiments were performed in accordance with Home Office regulations under an approved project license (PPL No: 60/4492).

### Gene Ontology Term Enrichment Analysis

Gene ontology (GO) term enrichment analysis was carried out using the GO.db (v3.2.2) and GOstats (v2.36.0) Bioconductor libraries in R; *P* values were generated using a hypergeometric test and adjusted for multiple testing (20).

### Statistical Analysis

Error bars represent SD. Statistical analyses were performed using the two-tailed Student’s *t* test or Gehan-Breslow Wilcoxon test. A *P* value of .05 or less was taken to be statistically significant.

Detailed information on all other methods can be found in the Supplementary Material (available online).

## Results

### Cellular Modeling of BCR-ABL-Independent Mechanisms of Resistance

**Figure 1.**
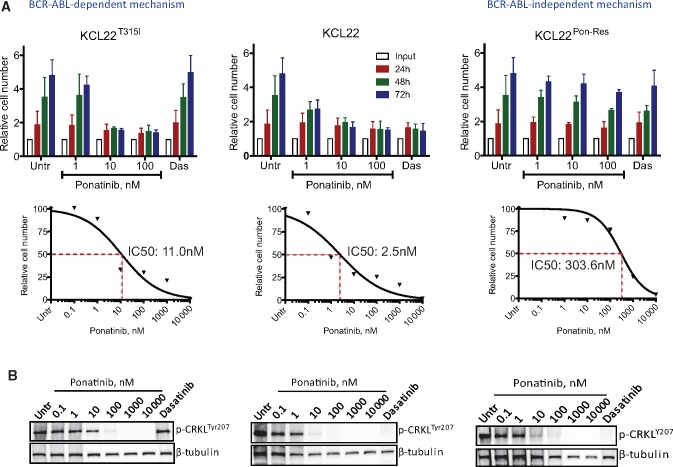
Proliferation of ponatinib-resistant chronic myeloid leukemia cells in the absence of BCR-ABL kinase activity. KCL22 (wild-type BCR-ABL), KCL22^T315I^, and KCL22^Pon-Res^ cells were cultured with or without (Untr) increasing concentrations of ponatinib and 150 nM dasatinib. Proliferation was measured by cell counting using a glass hemocytometer following 24, 48, and 72 hours of drug treatment, and IC50 values were calculated using GraphPad Prism software **(A)**. To assess for BCR-ABL activity, the levels of phosphorylation of CRKL were measured by immunoblot following four hours of drug treatment at varying concentrations of ponatinib and 150 nM dasatinib **(B)**. **Error bars** = SD. Two independent experiments were performed in triplicate. Untr = untreated.

Imatinib-resistant cell lines are often sensitive to more potent second-generation TKIs and/or ponatinib and are therefore not an ideal model to investigate acquired resistance to all available TKIs (Supplementary Figure 1, A–C, available online). Hence, we aimed to develop a ponatinib-resistant cell line with acquired BCR-ABL-independent resistance. KCL22 cells (human myeloid BC CML cell line) were grown in increasing concentrations of ponatinib for a prolonged period. Ponatinib-resistant (KCL22^Pon-Res^) clones continued to proliferate when exposed to 100 nM ponatinib (Figure 1A). Sequencing of the BCR-ABL kinase domain showed no kinase domain mutations (data not shown). Measuring tyrosine 207 phosphorylation of CRKL, a direct BCR-ABL substrate, revealed that BCR-ABL activity was inhibited to similar levels as in KCL22^T315I^ and parental KCL22 (Figure 1B).

### Transcriptional Response and mTORC1 Activity Following BCR-ABL Inhibition in KCL22^Pon-Res^ Cells

**Figure 2.**
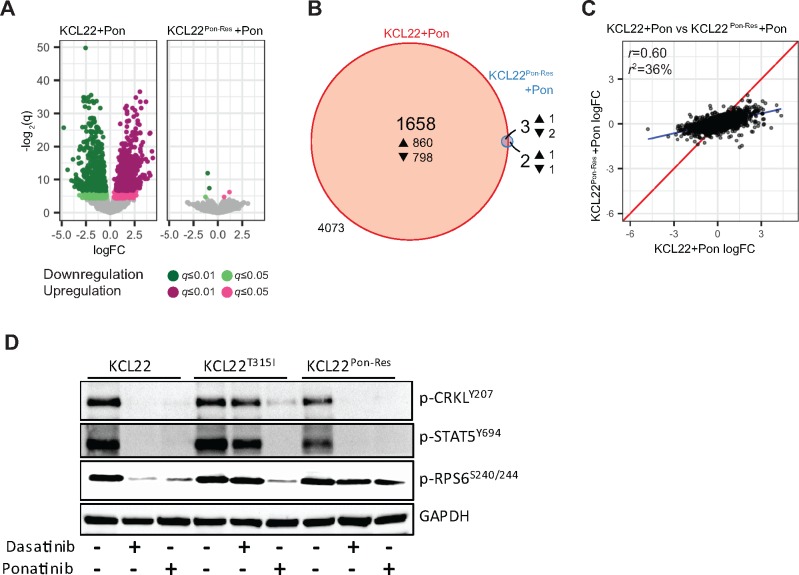
Transcriptional response and mTORC1 activity in ponatinib-resistant cells following BCR-ABL inhibition. KCL22 and KCL22^Pon-Res^ cells were cultured with or without 100 nM ponatinib for 24 hours and RNA harvested for RNA-seq. **A)** The transcriptional response of KCL22 and KCL22^Pon-Res^ cells is represented by Volcano plots (up- and downregulation are indicated by **magenta** and **green**, respectively; **light and dark colors** correspond to *q*-value thresholds of 0.05 and 0.01, respectively; statistically nonsignificant changes are colored **gray**). **B)** A proportional Venn diagram represents the overlap in statistically significant response to ponatinib (*q* ≤ 0.05) in both cell lines (4073 refers to the number of genes not changed). **C)** A direct comparison of the transcriptional response of all genes in both cell lines; identical expression is shown by the **red line**; the true linear relationship is indicated by the **blue line**. **D)** KCL22, KCL22^T315I^, and KCL22^Pon-Res^ cells were cultured ± 150 nM dasatinib or 100 nM ponatinib. Phosphorylation of CRKL, STAT5, and RPS6 was measured after 24 hours of drug treatment.

To investigate the potential mechanism(s) of resistance in KCL22^Pon-Res^ cells, parental KCL22 and KCL22^Pon-Res^ cells were treated with ponatinib to fully switch off BCR-ABL signaling (Supplementary Figure 2A, available online), and RNA was harvested for transcriptomic analysis. Of the 5736 gene transcripts that met the necessary expression thresholds in the RNA sequencing (RNA-seq) experiment, only 250 were differently expressed between the two cell lines (Supplementary Figure 2B, available online). Pathway enrichment analysis highlighted 42 potentially deregulated pathways (Supplementary Figure 2C, available online). More strikingly, while 1661 were differentially expressed following ponatinib-mediated BCR-ABL inhibition in the parental KCL22 cells, the same treatment had virtually no effect on the transcriptome of KCL22^Pon-Res^ cells (Figure 2, A and B). There was no correlation (*r* = 0.60) between the two cell lines in the transcriptional response of the 5736 genes to ponatinib (Figure 2C). This suggested that signaling pathways downstream of BCR-ABL (and normally inhibited by TKIs) remained active following BCR-ABL inhibition in KCL22^Pon-Res^ cells.

To investigate further the mechanism(s) of resistance of KCL22^Pon-Res^ cells, parental KCL22, KCL22^T315I^, and KCL22^Pon-Res^ cells were treated with dasatinib or ponatinib, and inhibition of targets downstream of BCR-ABL was measured. This revealed inhibition of CRKL and STAT5 phosphorylation, indicative of complete inhibition of BCR-ABL, but sustained phosphorylation of the translation regulator ribosomal protein S6 (RPS6) indicated activation of mTOR complex 1 (mTORC1) (21), a common downstream node on which multiple oncogenic signaling pathways converge (Figure 2D;Supplementary Figure 2A, available online).

### Drug Repurposing Screen in KCL22^Pon-Res^ Cells

**Figure 3.**
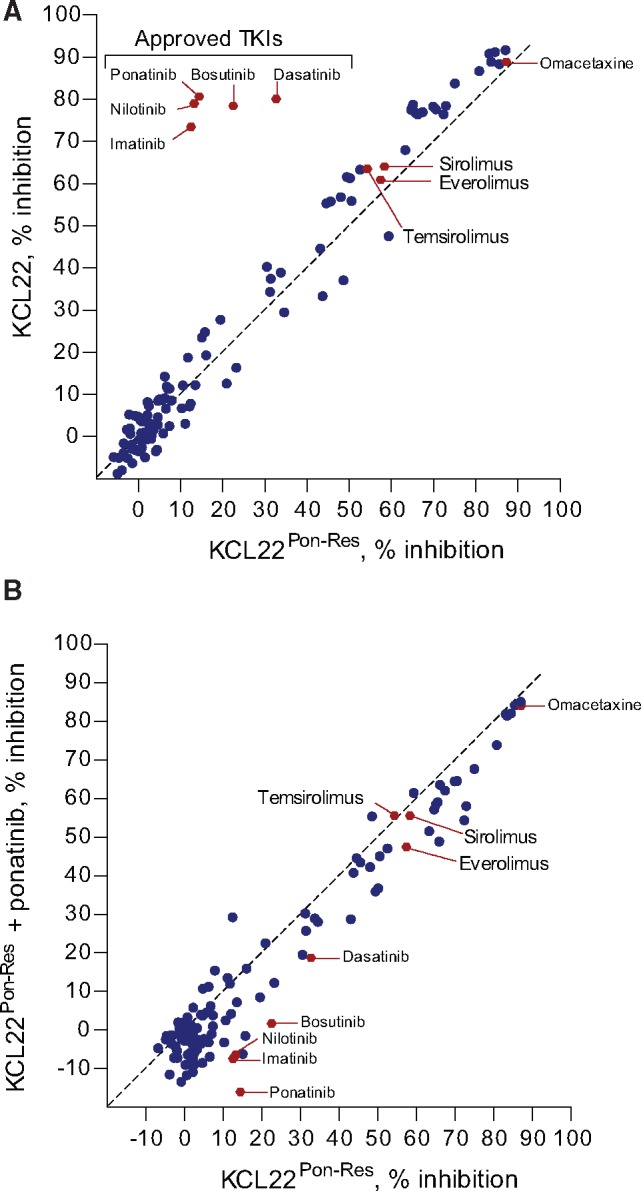
Sensitivity of ponatinib-resistant cells to allosteric mTORC1 inhibition. An approved oncology drug library was screened against KCL22 and KCL22^Pon-Res^ cells. Following 72 hours of 1 µM drug treatment, metabolic activity/proliferation was assessed using resazurin assay. Relative IC50 was calculated for each drug used, and a comparison was made between KCL22 and KCL22^Pon-Res^ cells **(A)** and between KCL22^Pon-Res^ cells cultured in the absence or presence of 100 nM ponatinib **(B).**

We next aimed to identify approved anticancer drug(s) with efficacy against TKI-resistant cells. We performed a screen using a validated oncogene drug library of 119 approved oncology drugs (Supplementary Table 1, available online). KCL22^Pon-Res^ cells were cultured alone or in combination with 100 nM ponatinib. The effect of additional drug exposure on cell survival was measured (omacetaxine mepesuccinate, a US Food and Drug Administration (FDA)–approved but nonselective and toxic inhibitor of total protein biosynthesis [22] was used as a control drug). This approach identified 36 drugs that were more effective in inhibiting proliferation of KCL22^Pon-Res^ cells when compared with all BCR-ABL-targeting TKIs tested (Supplementary Figure 3A, available online). Identified drugs included various conventional chemotherapeutic drugs and more specific kinase inhibitors. Comparison of drug sensitivity between parental KCL22 and KCL22^Pon-Res^ cells confirmed resistance of KCL22^Pon-Res^ cells to all FDA-approved BCR-ABL-targeting TKIs and demonstrated that all other drugs that are effective against parental KCL22 cells at 1 µM retained their activity against KCL22^Pon-Res^ cells (Figure 3A). Comparison of untreated and KCL22^Pon-Res^ cells grown in the presence of 100 nM ponatinib showed that single-agent treatment was similarly effective as when combined with complete BCR-ABL inhibition (Figure 3B). Subsequent target association analysis enriched for and highlighted microtubule, proteasome, and allosteric mTORC1 inhibitors (Supplementary Figure 3B, available online). However, these microtubule and proteasome inhibitors have known toxicities in the clinic. With IC50 levels for everolimus, sirolimus, and temsirolimus all below 200 nM (indicating on-target effect), regardless of whether used alone or in combination with ponatinib (Supplementary Figure 3, A–C, available online), we decided to focus our subsequent work on mTOR as a potential target for TKI resistance. This decision was also supported by the data shown in Figure 2B and Supplementary Figure 2 (available online), which suggest that sustained mTORC1 activity may support survival of KCL22^Pon-Res^ cells following TKI treatment.

### Sensitivity of KCL22^Pon-Res^ and TKI-Resistant Primary CML Cells to Catalytic mTOR Inhibitors

**Figure 4.**
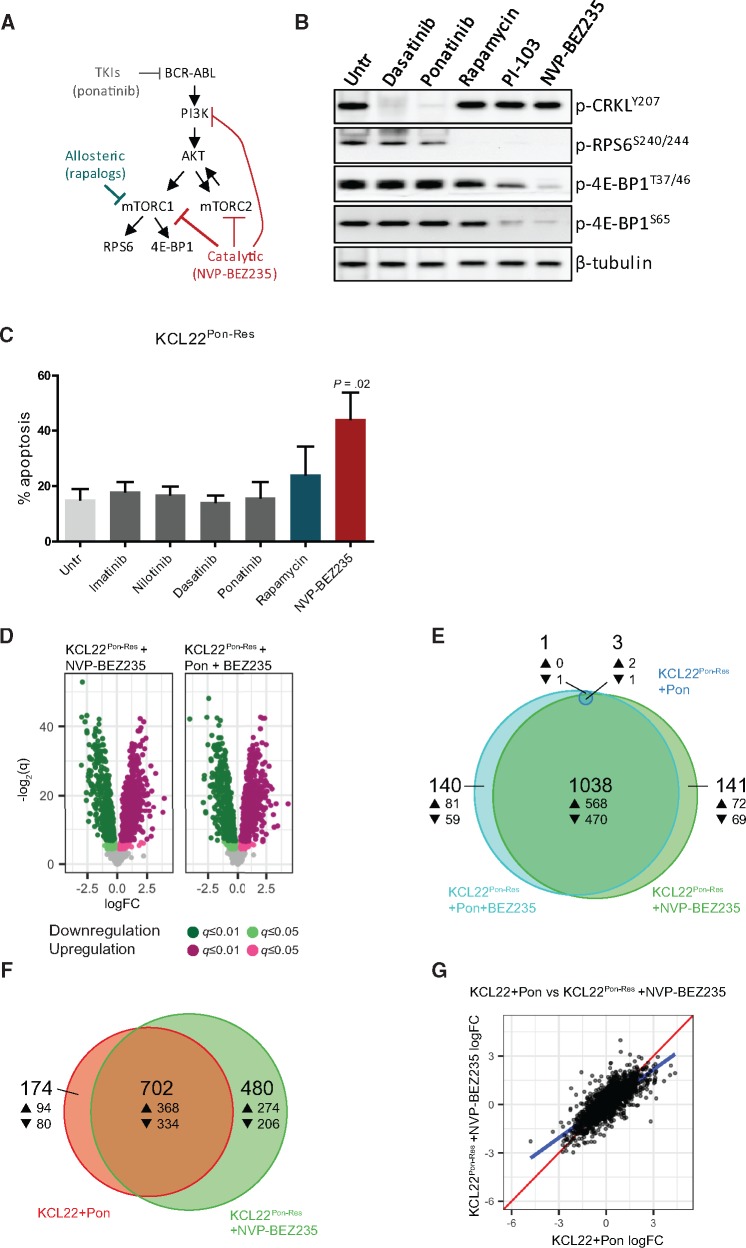
Transcriptional changes and levels of apoptosis in ponatinib-resistant cells following treatment with catalytic mTOR inhibitors. **A)** Schematic diagram demonstrating the activity of allosteric (**blue-green**) and catalytic (**red**) mTOR inhibitors. **B)** KCL22^Pon-Res^ cells were cultured with 150 nM dasatinib, 100 nM ponatinib, 10 nM rapamycin, 500 nM PI-103, or 100 nM NVP-BEZ235 or untreated (Untr). Phosphorylation of CRKL, RPS6, and 4E-BP1 was measured four hours following drug treatment. **C)** KCL22^Pon-Res^ cells were cultured ± 2 µM imatinib, 2 µM nilotinib, 150 nM dasatinib, 100 nM ponatinib, 10 nM rapamycin, or 100 nM NVP-BEZ235, and apoptosis was measured following 72 hours of drug treatment. **Error bars** = SD. Three independent experiments were performed. **D–G)** KCL22^Pon-Res^ cells were cultured ± 100 nM NVP-BEZ235, alone and in combination with 100 nM ponatinib for 24 hours, and RNA was harvested for RNA-seq. **D)** The transcriptional response of KCL22^Pon-Res^ cells to NVP-BEZ235 alone (**left**) and in combination with ponatinib (**right**) is represented by Volcano plots (up- and downregulation are indicated by **magenta** and **green**, respectively; **light and dark colors** correspond to *q*-value thresholds of 0.05 and 0.01, respectively; statistically nonsignificant changes are colored **gray**). **E)** A proportional Venn diagram represents the overlap in statistically significant response (*q* ≤ 0.05) to ponatinib alone (**dark blue**), NVP-BEZ235 alone (**green**), and the combination (**light blue**) in the KCL22^Pon-Res^ cells. **F)** A proportional Venn diagram represents the overlap in statistically significant response (*q* ≤ 0.05) to ponatinib in KCL22 cells (**red**) and NVP-BEZ235 in KCL22^Pon-Res^ cells (**green**). **G)** A direct comparison of the transcriptional responses of all 1718 genes to treatment common to both experiments; identical expression is shown by the **red line**, and the true linear relationship is indicated by the **blue line**. One independent experiment was performed in quadruplicate. Untr = untreated.

To date, sirolimus (rapamycin) and rapamycin analogues (allosteric mTORC1 inhibitors) (Supplementary Table 2, available online) have only shown modest efficacy in clinical trials (23). This is believed to be because they are incomplete, substrate-selective mTORC1 inhibitors (24). However, with the development of catalytic mTOR inhibitors, it is still hoped that mTOR represents a druggable target in malignancies driven by activation of the mTOR pathway. To confirm deeper mTORC1 inhibition with catalytic mTOR inhibitors, KCL22^Pon-Res^ cells were treated with PI-103 and its derivative NVP-BEZ235 (which inhibit both mTORC1 and mTORC2 and have activity against all PI3K isoforms [25,26]) and compared with rapamycin (Figure 4A). In line with previous studies, rapamycin had little effect on phosphorylation of 4E-BP1, whereas PI-103 and NVP-BEZ235 (using IC50 concentrations) led to reduction in 4E-BP1 phosphorylation, demonstrating more potent mTORC1 inhibition (Figure 4B). Seventy-two hours of drug treatment led to modest induction of apoptosis by rapamycin, with more extensive and statistically significant apoptosis observed following NVP-BEZ235 treatment (44.2[9.6]%, *P* = .02), whereas TKIs had no effect (Figure 4C). Similar effects were seen in colony forming cell (CFC) assay (data not shown). To test if these findings would replicate using different BCR-ABL-positive cell lines, we generated ponatinib-resistant BaF3 cells (BaF3^Pon-Res^), which were also highly sensitive to NVP-BEZ235, showing that sensitivity of ponatinib-resistant cells to mTOR inhibition is not restricted to the KCL22^Pon-Res^ cell line (Supplementary Figure 4A, available online). We then examined whether ponatinib-mediated BCR-ABL inhibition further enhanced the effect of NVP-BEZ235. In line with Figure 3B and Supplementary Figure 3C (available online), no increase was observed following ponatinib and NVP-BEZ235 combination over NVP-BEZ235 alone when apoptosis or CFC was measured (Supplementary Figure 4, B and C, available online).

Encouraged by these results and to translate our findings closer to the clinic, we compared the effect of ponatinib with various catalytic mTOR inhibitors on available progenitor cells derived from the BM of a patient who had failed to achieve complete cytogenetic response following first-, second-, and third-generation TKI treatments. Importantly, while ponatinib was ineffective, the catalytic mTOR inhibitors NVP-BEZ235 (26), Gedatolisib (27,28), Apitolisib (29,30), VS-5584 (31), and AZD8055 (32) all induced apoptosis over and above the ponatinib-treated arm (Supplementary Figure 5, available online).

To further understand the mechanism by which NVP-BEZ235 induced death, we performed RNA-seq on parental KCL22 and KCL22^Pon-Res^. Strikingly, while ponatinib had no effect on gene transcription in KCL22^Pon-Res^ cells (Figure 2, A and B), NVP-BEZ235 was sufficient to induce transcriptional changes on the same scale as ponatinib-treated parental KCL22 cells (Figure 4D, compare with Figure 2A). Further analysis showed that the majority of gene changes following ponatinib and NVP-BEZ235 combination treatment were accounted for in the NVP-BEZ235 single arm (Figure 4E). Additionally, comparison of transcriptional changes in ponatinib-treated KCL22 cells and NVP-BEZ235-treated KCL22^Pon-Res^ cells showed a high correlation (*r* = 0.78) in transcriptional effect, with the majority (∼80%) of statistically significant (*q* ≤ 0.05) changes following BCR-ABL inhibition in the parental cells also occurring in NVP-BEZ235-treated KCL22^Pon-Res^ cells (Figure 4, F and G). Substantial overlap in transcriptional changes was also shown when NVP-BEZ235-treated KCL22 cells were included in the analysis, demonstrating that targeting mTOR downstream of BCR-ABL rescues the impaired transcriptional response following BCR-ABL inhibition in TKI-resistant cells (Supplementary Figure 6A, available online). GO enrichment analysis of the differentially expressed genes showed that ponatinib treatment in KCL22 and NVP-BEZ235 treatment in KCL22^Pon-Res^ cells affected genes involved in the execution of apoptosis and DNA repair (Supplementary Figure 6Bi, available online). A parallel analysis demonstrated that NVP-BEZ235 treatment was additionally associated with changes in protein synthesis/mRNA translation (Supplementary Figure 6Bii, available online).

### The Effect of Catalytic mTORC1 Inhibition on Autophagy in KCL22^Pon-Res^ Cells 

**Figure 5.**
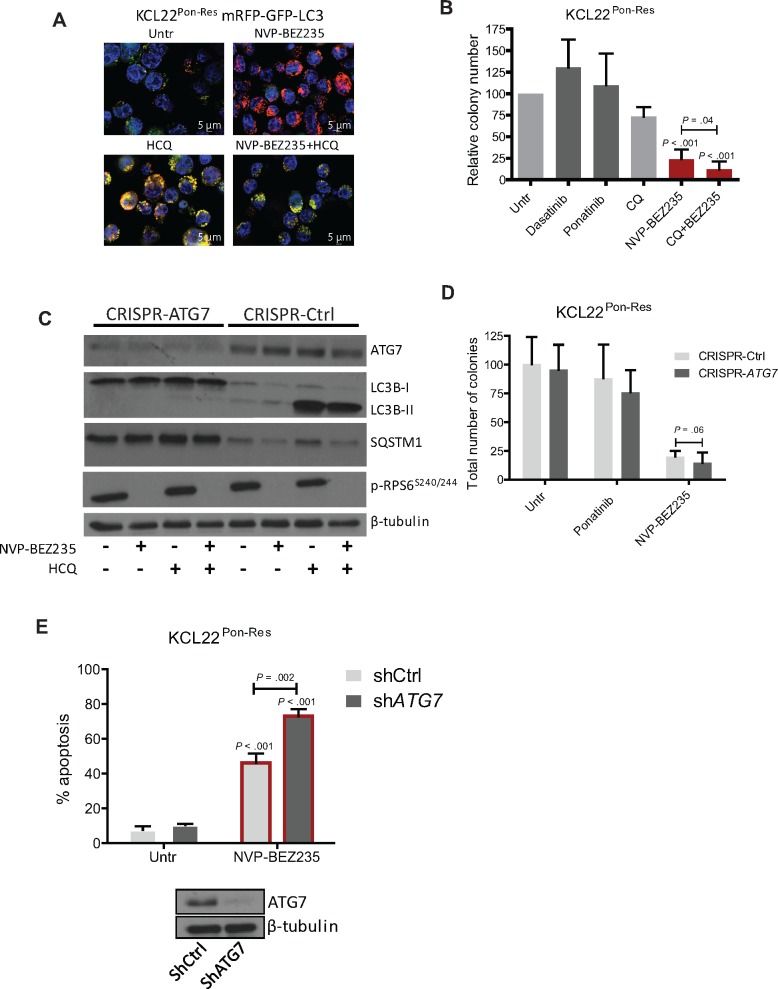
Autophagic response following mTOR inhibition in ponatinib-resistant cells. **A)** KCL22^Pon-Res^ cells expressing mRFP-GFP-LC3 were cultured ± 100 nM NVP-BEZ235 alone (t**op panel**) or in combination with 10 µM hydroxychloroquine (HCQ; **bottom panel**). **Scale bars** = 5 µm. Autophagy flow (**top panel**) and inhibition of autophagy flow (**bottom panel**) was visualized following 24 hours of drug treatment. **B)** KCL22^Pon-Res^ cells were cultured ± 150 nM dasatinib, 100 nM ponatinib, and 100 nM NVP-BEZ235 with and without chloroquine-mediated autophagy inhibition. Colony forming potential was measured following 72 hours of drug treatment. **C–E)** KCL22^Pon-Res^ cells were infected with lentivirus-expressing sgRNA **(C and D)** or shRNA-targeting **(E)***ATG7* or empty vector/scrambled (Scr) shRNA as control. Following knockdown, cells were treated with 100 nM ponatinib **(D)**, 100 nM NVP-BEZ235 **(C–E)** alone, or in combination with 10 µM HCQ **(C)**. **C and E)** Stable ATG7 knockdown, inhibition of autophagy (LC3-II and SQSTM1 levels), and mTORC1 activity were measured in puromycin-selected cells by immunoblot. Colony forming potential **(D)** or apoptosis **(E)** was measured following 72 hours of drug treatment. **Error bars** = SD. Statistical analysis was performed using the two-tailed Student’s *t* test. CQ = chloroquine; Untr = untreated.

mTORC1 not only regulates mRNA translation, but is also the master regulator of autophagy (macro-autophagy) (33,34). To assess autophagy flow, we generated KCL22^Pon-Res^ cell lines stably expressing fluorescence-tagged human LC3B (mRFP-GFP-LC3B) that enable different stages of autophagy to be visualized by fluorescence microscopy (35). The appearance of red/green puncta (yellow when overlapped) indicates autophagosomes, and as GFP is highly susceptible to the low pH within the lysosomes, a “red only” signal indicates autolysosomes. This process can be inhibited by hydroxychloroquine (HCQ), which inhibits autophagy at a late stage by preventing the fusion of autophagosomes and lysosomes, leading to build-up of yellow fluorescence. NVP-BEZ235 treatment increased puncta exhibiting a “red only” signal (autophagy flow complete) in KCL22^Pon-Res^ cells (Figure 5A). This indicates that NVP-BEZ235 induces autophagy flow, which can be effectively inhibited when combined with HCQ treatment.

Next, we investigated if autophagy induced by NVP-BEZ235 has a protective role. KCL22^Pon-Res^ cells were treated in combination with chloroquine (CQ)-mediated autophagy inhibition. While CQ treatment alone did not lead to statistically significant reduction in colony formation, it statistically significantly increased the cell death effect of NVP-BEZ235 (*P* = .04) (Figure 5B). Further combination experiments demonstrated that CQ and NVP-BEZ235 are synergistic in inhibiting proliferation of KCL22^Pon-Res^ cells when CQ is used at the 5–20 µM concentration range (combination index for NVP-BEZ235/CQ concentrations of 50 nM/5 µM, 100 nM/10 µM, 150 nM/15 µM and 200 nM/20 µM were 0.18, 0.60. 0.50, and 0.52, respectively) (Supplementary Figure 7, A and B, available online). CRISPR-Cas9 and RNAi techniques were employed to test if the specific inhibition of autophagy would enhance NVP-BEZ235-induced death. Initially, *ATG7*, an E1-like enzyme and essential autophagy gene required for LC3 lipidation, was targeted using CRISPR-Cas9. *ATG7* knockdown inhibited LC3B-II formation and autophagy, measured by a decrease in LC3B-II levels and an increase in the autophagy substrate SQSTM1/p62 (36,37) (Figure 5C), and indeed sensitized cells to death following NVP-BEZ235-mediated mTOR inhibition (*P* = .06) (Figure 5D). Similarly, RNAi-mediated *ATG7* knockdown statistically significantly increased the effect of the NVP-BEZ235 on apoptosis, confirming that autophagy plays a protective role in KCL22^Pon-Res^ cells following inhibition of mTORC1 (*P* = .002) (Figure 5E).

### The Effect of Pharmacological Inhibition of Autophagy and NVP-BEZ235 Treatment In Vivo and in TKI-Resistant Primary CML Cells

**Figure 6.**
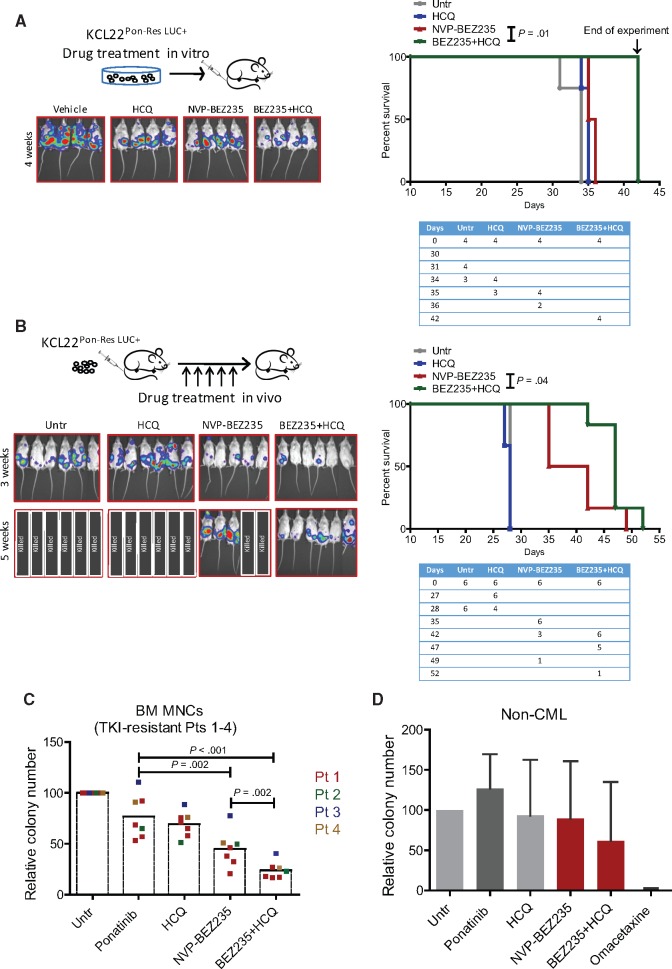
Sensitivity of xenografted ponatinib-resistant cells and primary chronic phase TKI-resistant chronic myeloid leukemia cells to NVP-BEZ235 and hydroxychloroquine (HCQ)-mediated autophagy inhibition. **A)** KCL22^Pon-Res^ cells were labeled with firefly luciferase and treated ex vivo with 100 nM NVP-BEZ235, alone and in combination with 10 µM HCQ. Seventy-two hours following drug treatment, cells were transplanted intravenously into sublethally irradiated NSG mice (four mice per group, two independent experiments). Thirty minutes after the transplant, the mice were injected with D-luciferin substrate to ensure the success of the transplantation and the cell viability. Leukemic progression was measured weekly by luciferase bio-imaging (**left**). Overall survival was monitored by Kaplan-Meier analysis (**right**). A table showing the number of mice at risk is shown below the graph. **B)** Firefly luciferase labeled KCL22^Pon-Res^ cells were transplanted intravenously into NSG mice (five to six mice per group, two independent experiments). Mice were then treated with NVP-BEZ235 (45 mg/kg, oral gavage), HCQ (60 mg/kg, intraperitoneal injection), and the combination for up to five weeks. Leukemia progression and overall survival were measured by luciferase bio-imaging (**left**) and Kaplan-Meier analysis (**right**), respectively. A table showing the number of mice at risk is shown below the graph. Untr = untreated. **C)** Bone marrow (BM)–derived mononuclear cells (MNCs) from four TKI-resistant patients (Pts 1–4) were cultured in SFM supplemented with PGF and treated with 100 nM ponatinib, 100 nM NVP-BEZ-235 alone, or in combination with HCQ-mediated autophagy inhibition for 72 hours. Survival of progenitor cells was measured by colony forming cell (CFC) assay. Each **dot** represents average of two to three technical replicates. **D)** BM cells were collected on four occasions from patient No. 1 (Pt 1) during the period of 2013–2016. Ph-negative CD34^+^ cells (n = 4) were cultured in SFM supplemented with PGF and treated with 100 nM ponatinib, 100 nM NVP-BEZ-235, 10 µM HCQ, 10 nM omacetaxine, and the combination of NVP-BEZ-235 and HCQ. Survival of progenitor cells was measured by CFC assay following 72 hours of drug treatment. **Error bars** = SD. Statistical analyses were performed using the Gehan-Breslow Wilcoxon test **(A and B)** or the two-tailed Student’s *t* test **(C and D)**. CML = chronic myeloid leukemia; HCQ = hydroxychloroquine; Untr = untreated.

We next tested whether mTOR inhibition can interfere with leukemia initiation when combined with pharmacological autophagy inhibition. KCL22^Pon-Res^ cells were labeled with lentiviral luciferase and treated ex vivo with NVP-BEZ235, HCQ, and the combination. Following drug treatment, cells were injected into NSG mice, which were then monitored weekly by luciferase bio-imaging. At week 4, there was a marked delay in leukemia development in mice engrafted with cells treated with the combination (Figure 6A). The combination treatment also statistically significantly prolonged overall survival of xenografted NSG mice when compared with NVP-BEZ235 single treatment (*P* = .01) (Figure 6A).

To test the tolerability and efficacy of this drug combination, we injected luciferase-expressing KCL22^Pon-Res^ cells into NSG mice. Following evidence of engraftment, the xenografted mice were treated for up to five weeks. By week 3, bio-imaging showed leukemia development in untreated and HCQ-treated mice (Figure 6B). By five weeks, all untreated and HCQ-treated mice had to be killed, together with two out of five mice treated with NVP-BEZ235 alone. Mice showed few signs of toxicity, and the combination statistically significantly extended the survival of mice when compared with NVP-BEZ235 single treatment (median survival NVP-BEZ235 vs NVP-BEZ235+HCQ: 38.5 days vs 47.0 days, *P* = .04) (Figure 6B).

Finally, we compared the effect of NVP-BEZ235 in combination with HCQ with ponatinib on cells derived from the BM of four patients who had failed to achieve cytogenetic response following first-, second-, or third-generation TKI treatments (Supplementary Table 3, available online). Importantly, NVP-BEZ235 had a greater effect on survival of progenitor cells in these patients (Figure 6C). HCQ treatment statistically significantly enhanced the effect of NVP-BEZ235 (NVP-BEZ235 vs NVP-BEZ235+HCQ: 45.0[17.9]% vs 24.0[8.4]%, *P* = .002). Colony polymerase chain reaction and fluorescence in situ hybridization confirmed that the vast majority of cells were Ph positive (data not shown). To test the effect of this combination on normal cells, non-CML cells (derived from patients with Ph-negative, nonmyeloid hematological malignancies) were treated with ponatinib, NVP-BEZ235 alone, and in combination with HCQ, and compared with a cytotoxicity of 10 nM omacetaxine treatment. This revealed that while omacetaxine substantially affected the CFC potential of normal progenitor cells, the combination of NVP-BEZ235 and HCQ had only a minimal effect (Figure 6D).

## Discussion

Despite the promising results in the PACE trial, where ponatinib induced rapid and durable responses in CP-CML patients, it is associated with considerable cardiovascular toxicity that may be dose-dependent. Additionally, a proportion of patients taking ponatinib already have or will develop BCR-ABL-independent mechanisms of resistance, and therefore fail ponatinib treatment. Therefore, it is hoped that this patient population that currently experiences rare response to TKI treatment and very short survival may share an alternative drug target that can be inhibited with a novel compound. We, therefore, for the first time, generated a ponatinib-resistant cell line, which developed BCR-ABL-independent activation of mTOR. This afforded us a unique opportunity to search for drugs that are effective against ponatinib-resistant CML cells. Our screen and subsequent testing of catalytic mTOR inhibitors revealed that NVP-BEZ235 had increased potency in inhibiting mTORC1 in ponatinib-resistant cells. This correlated with potent transcriptional response and induction of apoptosis, in agreement with previous studies where catalytic mTOR inhibitors, such as OSI-027 and PP242 (or dual PI3K/mTOR inhibitors, such as PI-103 and NVP-BEZ235), have been shown to prevent expansion of Ph-positive acute lymphoblastic leukemia cells in vivo (38), to sensitize CML cells to nilotinib (39,40), and to be effective in targeting CML cells in vitro (41,42). Critically, we also showed that NVP-BEZ235 was more effective than ponatinib against available primary cells obtained from heavily pretreated TKI-resistant CML patients. Although further investigation will be required to confirm the exact mechanism of resistance in each patient (requires optimized protocols for rare BM-aspirated cells), this provides a rationale for testing catalytic mTOR inhibitors in the clinic for patients who do not respond to BCR-ABL inhibitors.

A phase I dose-finding study of NVP-BEZ235 is ongoing in patients with relapsed or refractory acute leukemia (NCT01756118). Based on a phase I trial in patients with advanced solid tumors, the recommended dose for NVP-BEZ235 is 300 mg twice daily (BID), which is still expected to inhibit mTORC1/2 according to pharmacodynamic data (43). However, recent results from a phase II trial for patients with everolimus-resistant pancreatic neuroendocrine tumors (NCT01658436) show that many patients experience toxicities on 300 mg BID, although this may also reflect the fragility of the heavily pretreated patients with this aggressive cancer (44).

It is clear that the depth of response to TKI is the major driver for sustained remissions, hence the need to rapidly reduce overall leukemic cell burden and ideally to reduce the numbers of BM-located cells (45). We showed that HCQ treatment (a nonspecific autophagy inhibitor that is being tested in more than 30 active clinical trials [46]) inhibited autophagy flow and enhanced death following NVP-BEZ235, both in vitro and in a xenograft model of CML. Importantly, we also showed that genetic autophagy inhibition sensitized CML cells to death, indicating that the main additive effect of HCQ was due to a block in the autophagy process, but not off target effect.

A recent phase I trial in patients with advanced solid tumors and melanoma shows that HCQ is safe and tolerable and has some antitumor activity when used in combination with temsirolimus-mediated mTOR inhibition (47). Although our results on normal blood cells suggest that HCQ and NVP-BEZ235-mediated mTOR inhibition may be tolerated with regards to myelosuppression, results from phase I trials are awaited for different innovative catalytic mTOR inhibitors such as Gedatolisib, Apitolisib, VS5584, and AZD8055 (all in phase I; Apitolisib in phase II). The outcome of these studies may determine the most suitable catalytic mTOR inhibitor (in terms of efficacy and tolerability) to be taken forward for combination studies.

Given that the mechanism(s) of resistance to TKIs may vary from patient to patient, potential limitations of this study should be considered. First, the in vitro studies of primary CML cells in response to the catalytic mTOR inhibitor(s) presented here were confined to a relatively small number of TKI-resistant CML patients’ samples. Additionally, HCQ is nonspecific autophagy inhibitor, and development of more specific and/or more potent autophagy inhibitors might be required to inhibit autophagy in BM-located cells in CML patients.

We conclude that catalytic mTOR inhibitors may be effective for patients with BCR-ABL-independent resistance and that pharmacological autophagy inhibition will further enhance their efficacy. This is particularly important for this heavily pretreated population because treatment options for patients who fail all currently available TKIs, including ponatinib, are very limited.

## Funding

This work was supported by Medical Research Council (G0600782 and G0900882, *CHOICES*, ISCRTN No. 61568166), the Kay Kendall Leukaemia Fund (KKL404 and KKL501), Leuka, Glasgow Experimental Cancer Medicine Centre, which is funded by Cancer Research UK and the Chief Scientist's Office (Scotland), Cancer Research UK Glasgow Centre (C596/A18076) and the BSU facilities at the Cancer Research UK Beatson Institute (C596/A17196), Scottish Universities Life Science Alliance (MSD23_G_Holyoake-Chan), Scottish National Blood Transfusion Service, Cancer Research UK programme funding (C11074/A11008), the Howat Foundation and Friends of Paul O’Gorman (flow cytometry support). GVH is a Kay Kendall Leukaemia Fund (KKLF) Intermediate Research Fellow (KKL698)/Leadership Fellow/John Goldman Fellow. LH is a KKLF Intermediate Research Fellow (KKL1148)/John Goldman Fellow. BC is supported, in part, by National Cancer Institute grant CA95111. PS is funded by the Brain Tumour Charity, Cancer Research UK (CRUK) and Association for International Cancer Research (AICR) and is supported by the National Institute for Health Research University College London Hospitals Biomedical Research Centre. BJD is funded by the Howard Hughes Medical Institute and National Institutes of Health grant R37CA065823.

## Notes

Affiliations of authors: Glasgow Polyomics (GH, PH), Wolfson Wohl Cancer Research Centre (RM, PB, GVH), Paul O’Gorman Leukaemia Research Centre (LEMH, AM, AH, TLH), Institute of Cancer Sciences, University of Glasgow, Glasgow, UK; Scottish National Blood Transfusion Service, Gartnavel General Hospital, Glasgow, UK (EKA); Cancer Research UK, Beatson Institute, Garscube Estate, Glasgow, UK (KH, DJ, JO, KMR, ES); Faculty of Health and Medical Sciences, University of Adelaide, Adelaide, Australia and Imperial College, London, UK (JVM); Strathclyde Institute of Pharmacy and Biomedical Sciences, University of Strathclyde, Glasgow, UK (EC); Hématologie Clinique 1G, Centre Hospitalier Lyon Sud, Pierre Bénite, France (VMS, FEN); Division of Hematology and Medical Oncology, Oregon Health and Science University, Knight Cancer Institute, Portland, OR (BJD); Institute of Translational Medicine, Department of Molecular and Clinical Cancer Medicine, University of Liverpool, UK (REC); Department of Haematology, Milton Keynes Hospital NHS Foundation Trust, Milton Keynes, UK (SM); Institute of Molecular, Cell and Systems Biology, College of Medical, Veterinary and Life Sciences, University of Glasgow, UK (PH); Samantha Dickson Brain Cancer Unit, UCL Cancer Institute, Paul O'Gorman Building, London, UK (PS); Department of Cancer Biology, Kimmel Cancer Center, Thomas Jefferson University, Philadelphia, PA (BC).

The funders had no role in the design of the study; the collection, analysis, or interpretation of the data; the writing of the manuscript; or the decision to submit the manuscript for publication.

TLH has previously received research support from Bristol-Myers Squibb and Novartis. FEN is a consultant for Novartis, ARIAD, and Pfizer, has received research grants from Novartis, and has received speakers fees from Novartis, Bristol-Myers Squibb, ARIAD, and Pfizer. BJD is currently principal investigator or co-investigator on Novartis, Bristol-Myers Squibb, and ARIAD clinical trials.

The authors would like to dedicate this work to the memory of Prof. Tessa Holyoake, who was an inspiration to us all.

The next-generation sequencing was performed by Glasgow Polyomics and supported by the Wellcome Trust (105614/Z/14/Z). Firefly luciferase vector (pLenti CMV Puro LUC) was kindly provided by Mike Olson. mRFP-GFP-LC3 was kindly provided by Tamotsu Yoshimori. We thank A. Michie and K. Dunn for assisting with in vivo work (MRC/AstraZeneca project grants: Ref: MR/K014854/1), the National Health Service GGC Bio-repository Unit, Paolo Gallipoli and Susan Rhodes for collection of normal and TKI-resistant patient samples, UK Haematologists, and patients with chronic myeloid leukemia. We thank Bristol-Myers Squibb and ARIAD Pharmaceuticals for providing dasatinib and ponatinib, respectively.

Contributions: GVH, RM, and TLH wrote the manuscript. GVH, PS, BC, and TLH designed experiments. GVH, RM, EA, AM, PB, and ES performed experiments and interpreted data. PH, GH, and LEMH performed experiments and analyzed RNA-seq data. JOP, JM, EC, KMR, VMS, SM, FEN, and BJD provided reagents and materials. PS, BC, and TLH reviewed the manuscript.

## Supplementary Material

Supplementary Tables and Figures
