# Supplementary material for: Targeting BCR-ABL-Independent TKI Resistance in Chronic Myeloid Leukemia by mTOR and Autophagy Inhibition
Source: J Natl Cancer Inst. 2017 Nov 20;110(5):467–78. doi: 10.1093/jnci/djx236 (PMC5946859; doi:10.1093/jnci/djx236)
Supplement: Supplementary Tables and Figures [file djx236_supp.pdf]

SUPPLEMENTARY MATERIALS

Targeting BCR-ABL Independent TKI Resistance in Chronic Myeloid Leukaemia by mTOR and Autophagy Inhibition

Rebecca Mitchell,[1] Lisa E. M. Hopcroft,[2] Pablo Baquero,[1] Elaine K. Allan,[3] Kay Hewit,[4] Daniel James,[4] Graham Hamilton,[5] Arunima Mukhopadhyay,[2] Jim O'Prey,[4] Alan Hair,[2] Junia V. Melo,[6] Edmond Chan,[7] Kevin M. Ryan,[4] Véronique Maguer-Satta,[8] Brian J. Druker,[9] Richard E. Clark,[10] Subir Mitra,[11] Pawel Herzyk,[5,12] Franck E. Nicolini,[8] Paolo Salomoni,[13] Emma Shanks,[4] Bruno Calabretta,[14] Tessa L. Holyoake,[2] and G. Vignir Helgason[1*]

[1]Wolfson Wohl Cancer Research Centre, Institute of Cancer Sciences, University of Glasgow, G61 1QH, UK; [2]Paul O'Gorman Leukaemia Research Centre, Institute of Cancer Sciences, University of Glasgow, Glasgow, G12 0ZD, UK; [3]Scottish National Blood Transfusion Service, Gartnavel General Hospital, Glasgow, G12 0XB, UK; [4]Cancer Research UK, Beatson Institute, Garscube Estate, Glasgow, G61 1BD, UK; [5]Glasgow Polyomics, Wolfson Wohl Cancer Research Centre, University of Glasgow, G61 1QH, UK; [6]Faculty of Health and Medical Sciences, University of Adelaide, Adelaide, Australia and Imperial College, London, UK; [7]Strathclyde Institute of Pharmacy and Biomedical Sciences, University of Strathclyde, Glasgow, G4 0RE, UK; [8]Hématologie Clinique 1G, Centre Hospitalier Lyon Sud, Pierre Bénite, France; [9]Division of Hematology and Medical Oncology, Oregon Health & Science University, Knight Cancer Institute, Portland, Oregon, USA; [10]Institute of Translational Medicine, Department of Molecular and Clinical Cancer Medicine, University of Liverpool, L69 3BX, UK; [11]Department of Haematology, Milton Keynes Hospital NHS Foundation Trust, Milton Keynes, MK6 5LD, UK; [12]Institute of Molecular, Cell & Systems Biology, College of

Medical, Veterinary & Life Sciences, University of Glasgow, G12 8QQ, UK; [13]Samantha Dickson Brain Cancer Unit, UCL Cancer Institute, Paul O'Gorman Building, London, WC1E 6BT, UK; [14]Department of Cancer Biology, Kimmel Cancer Center, Thomas Jefferson University, Philadelphia, Pennsylvania, USA.

[*]Correspondence to: G. Vignir Helgason, Wolfson Wohl Cancer Research Centre, Institute of Cancer Sciences, University of Glasgow, G61 1QH, UK Vignir.Helgason@Glasgow.ac.uk.

Tel: +44 (0) 141 330 7245

Supplemental Methods

In vitro cell culture

BM-derived MNCs from TKI-resistant patients and non-CML $CD34^+$ cells were cultured in serum-free medium (SFM), supplemented with a physiological growth factor (PGF) cocktail as previously described (1). KCL22, $BaF3^{WT}$ (expressing WT p210 BCR-ABL), and ponatinib-resistant derivative cells ($KCL22^{Pon\text{-}Res}$ and $BaF3^{Pon\text{-}Res}$) were cultured in RPMI 1640 medium, supplemented with 1% penicillin/streptomycin, 1% L-glutamine, and 10% FCS (all Invitrogen). $KCL22^{Pon\text{-}Res}$ and $BaF3^{Pon\text{-}Res}$ cells were generated by culturing parental cells in increasing concentrations of ponatinib.

Drug Stock Solutions

Dasatinib (Bristol-Myers Squibb, Princeton, NJ, USA), ponatinib (ARIAD Pharmaceuticals, Cambridge, MA, USA), NVP-BEZ235 (LC Laboratories, MA, USA), PP242 (Sigma-Aldrich), gedatolisib, apitolisib, VS5584, AZD8055 (all Selleckchem, TX, USA), PI-103 (Merck KGaA, Darmstadt, Germany) and omacetaxine mepesuccinate (Chemgenex) were prepared in DMSO and stored at −20°C. Rapamycin (Sigma-Aldrich) was prepared in EtOH and stored at −20°C. HCQ (Sigma-Aldrich) was prepared in PBS and stored at −20°C. CQ (Sigma-Aldrich) was prepared in water and stored at 4°C.

Drug screening

An oncology drug library (NCI DTP Approved Oncology Drugs) was screened in $KCL22^{WT}$ and $KCL22^{Pon\text{-}Res}$ cells. Internal plate controls were included to allow statistical assessment of screening performance, namely vehicle control (DMSO), a lethality control (1µM omacetaxine), and a positive control (100nM ponatinib). Cells were plated at 25000 cpw (+/-

100nM ponatinib) using an XRD automated reagent dispenser (FluidX). Drugs were added to cell plates at a final concentration of 0.01, 0.1, 1 and 10µM using an automated liquid handling system (JANUS, Perkin Elmer). Drug effects were quantified using resazurin/resorufin conversion assay and quantifying fluorescence (535ex, 590em, EnVision multiplate reader, Perkin Elmer). Relative SoF was calculated for each drug by comparing the effect to lethal control arm.

RNA-seq and analysis

RNA was extracted using an RNeasy Mini kit (Qiagen, Cat no. 74104) as per the manufacturer's instructions followed by RNA enrichment using polyA selection. The sequence library was created using the TruSeq RNA Library Prep version 2 kit, to polyA select mRNA. The library insert size was ~300bp. The libraries were sequences on the Illumina NextSeq™500 platform with ~50 million paired end reads per sample. The sequences were trimmed using cutadapt v 1.0 (2) (quality value=25, minimum read length=56). The resulting reads were aligned to the human genome GRCh37 (p13) using HiSat2 v 2.0.4 (3) and mapped to Ensembl gene IDs. Genome coverage statistics were calculated by genomecov (bedtools v2.26.0). The number of reads per gene was quantified using htseq-count v 0.6.0 (4). Exonic gene lengths for calculation of RPKM values were obtained via the corresponding GTF file; genes with missing length information or <7 CPM (counts per million) were removed from further analysis. Count data were normalised by TMM (5) and VOOM (6) (as implemented in the Bioconductor packages edgeR v3.12.1 and limma v3.26.9 using R v3.2.2). Limma (6) was used to compare transcript abundance across treatment arms and generate logFC and adjusted p-values (q) (7). Proportional Venn diagrams were generated using Vennerable (v3.0) in R. Pearson correlation coefficients were calculated in R.

MSigDB enrichment analysis

Expression data for MSigDB v4.0 C2 (curated gene sets) signatures were summarised using GSVA (v1.24.1) (MSigDB obtained as XML from the Broad Institute, http://software.broadinstitute.org/gsea/msigdb). Limma (6) was used to calculate differential expression of these signatures and generate logFC and adjusted p-values (q) (7).

Western blotting

Western blotting was performed as per standard protocols (8) using antibodies against LC3B, β-tubulin, p-CRKL, p-STAT5, GAPDH, p-RPS6, p-4E-BP1, SQSTM1 (Cell Signalling) and ATG7 (Epitomics-Abcam, Burlingame, CA, USA) in 1/1000 dilution.

Autophagy monitoring by fluorescence

KCL22$^{Pon\text{-}Res}$ cells expressing mRFP-GFP-LC3 were fixed and washed twice with PBS before air drying and counter staining with DAPI (Vectashield) and visualised using a Zeiss Imager M1 AX10 fluorescence microscope with AxioVision 3D deconvolution software.

Apoptosis and CFC assay

Cells were resuspended in a solution made up of; 2.5μL annexin-V-APC (BD, Cat no. 550475) and 2.5μL 7-AAD (BD, Cat no. 559925) in 45μL 1x HBSS binding buffer (Thermo Fisher Scientific, Gibco Cat no. 14025100), incubated for 20 minutes in the dark and analysed by flow cytometry (Becton Dickinson FACSVerse). Data was analysed using FlowJo software. CFC assay was performed as previously described (1).

Virus production and cell transduction

For virus production, HEK293FT (Invitrogen) were transiently transfected with indicated plasmids by calcium phosphate method as previously described (1). Lentiviruses were produced with the addition of pCMV-VSV-G (envelope plasmid) and psPAX2 packaging constructs. mRFP-GFP-LC3 was cloned into the pWzl Hygro retroviral vector. KCL22$^{Pon\text{-}Res}$ cells were transduced with lentivirus expressing firefly luciferase (pLenti CMV Puro LUC; Addgene #17477), mRFP-GFP-LC3 or vector expressing Cas9 and stable cell lines generated by antibiotic selection. sgRNA sequences for CRISPR/Cas9-mediated deletion of ATG7 were designed using the MIT CRISPR design tool (http://crispr.mit.edu/). The following guide sequences was selected: 5′-GAAGCTGAACGAGTATCGGC-3′ and cloned into the LentiCRISPRv2 vector (Addgene #52961) according to the Zhang lab protocol. Knockdown was performed with pLKO.1 transfer vector containing verified shRNA specific for human *ATG7* (TRCN0000007584; Sigma-Aldrich) or non-targeting scrambled hairpin as control.

Synergism measurement

The synergistic effect of NVP-BEZ235 and CQ was predicted by the Chou–Talalay medium-effect method using CompuSyn software (ComboSyn, Inc. Paramus, NJ, 2005). A constant molar ratio of 1:100 of NVP-BEZ235: CQ was used based on the relative activities of both agents determined in previous experiments. The effect of the drugs was assessed by counting the viable cells after 72h of treatment using Casy TT Cell Counter and Analyser (Innovatis, Roche Applied Science). The concentrations used were from 25 to 200nM of NVP-BEZ235 and from 2.5 to 20µM for CQ. A combination index of less than 1 indicates synergism; more than 1, antagonism; and 1, additive effect.

Bioluminescent *in vivo* imaging

Human KCL22$^{Pon\text{-}Res}$ cells, labeled with lentiviral firefly luciferase, were injected via tail vein into 8-12 week old female NOD/SCID/IL-2Rγ-/- (NSG) recipient mice (1×10^6 cells per mouse) (9). 30 minutes later the mice were injected intraperitoneally with D-luciferin (3mg per mouse, Perkin Elmer, Cat no. 122799) and analysed by luciferase bio-imaging via an IVIS Spectrum In Vivo Imaging System (PerkinElmer, Cat no. 124262), to measure the efficiency of transplantation. The engraftment of these cells was subsequently measured weekly.

***In vivo* drug treatment**

Mice were treated with vehicle control (citrate buffer or NMP/PEG300 (1:10)), HCQ (60mg/kg, intraperitoneally once daily, Sigma, Cat no. 1327000), NVP-BEZ235, (40mg/kg, oral gavage once daily, LC Labs, Cat no. N-4288), or the combination of NVP-BEZ235/HCQ.

References

1. Hamilton A, Helgason GV, Schemionek M, *et al.* Chronic myeloid leukemia stem cells are not dependent on Bcr-Abl kinase activity for their survival. Blood 2012;119(6):1501-10.
2. Martin M. Cutadapt removes adapter sequences from high-throughput sequencing reads. EMBnetjounal 2011;17:10-12.
3. Kim D, Langmead B, Salzberg SL. HISAT: a fast spliced aligner with low memory requirements. Nat Methods 2015;12(4):357-60.
4. Anders S, Pyl PT, Huber W. HTSeq--a Python framework to work with high-throughput sequencing data. Bioinformatics 2015;31(2):166-9.
5. Robinson MD, Oshlack A. A scaling normalization method for differential expression analysis of RNA-seq data. Genome Biol 2010;11(3):R25.
6. Law CW, Chen Y, Shi W, *et al.* voom: Precision weights unlock linear model analysis tools for RNA-seq read counts. Genome Biol 2014;15(2):R29.
7. Benjamini Y, Hochberg Y. Controlling the False Discovery Rate: A Practical and Powerful Approach to Multiple Testing. Journal of the Royal Statistical Society 1995;57(1):289-300.
8. Copland M, Hamilton A, Elrick LJ, *et al.* Dasatinib (BMS-354825) targets an earlier progenitor population than imatinib in primary CML but does not eliminate the quiescent fraction. Blood 2006;107(11):4532-9.
9. Ishikawa F, Yasukawa M, Lyons B, *et al.* Development of functional human blood and immune systems in NOD/SCID/IL2 receptor {gamma} chain(null) mice. Blood 2005;106(5):1565-73.
10. Mahon FX, Deininger MW, Schultheis B, *et al.* Selection and characterization of BCR-ABL positive cell lines with differential sensitivity to the tyrosine kinase inhibitor STI571: diverse mechanisms of resistance. Blood 2000;96(3):1070-9.
11. Donato NJ, Wu JY, Stapley J, *et al.* BCR-ABL independence and LYN kinase overexpression in chronic myelogenous leukemia cells selected for resistance to STI571. Blood 2003;101(2):690-8.

Supplementary Tables

Supplementary Table 1. List of FDA-approved oncology drugs in drug library

Abiraterone
Afatinib
Allopurinol
Altretamine
Amifostine
Aminolevulinic acid hydrochloride
Anastrozole
Arsenic trioxide
Axitinib
Azacitidine
Belinostat
Bendamustine hydrochloride
Bleomycin sulfate
Bortezomib
Bosutinib
Busulfan
Cabazitaxel
Cabozantinib
Capecitabine
Carboplatin
Carfilzomib
Carmustine
Celecoxib
Ceritinib
Chlorambucil
Cisplatin
Cladribine
Clofarabine
Crizotinib
Cyclophosphamide
Cytarabine hydrochloride
Dabrafenib mesylate
Dacarbazine
Dactinomycin
Dasatinib
Daunorubicin hydrochloride
Decitabine
Dexrazoxane
Docetaxel
Doxorubicin hydrochloride

Enzalutamide
Epirubicin hydrochloride
Erlotinib hydrochloride
Estramustine phosphate sodium
Etoposide
Everolimus
Exemestane
Floxuridine
Fludarabine phosphate
Fluorouracil
Fulvestrant
Gefitinib
Gemcitabine hydrochloride
Hydroxyurea
Ibrutinib
Idarubicin hydrochloride
Idelalisib
Ifosfamide
Imatinib
Imiquimod
Irinotecan hydrochloride
Ixabepilone
Lapatinib
Lenalidomide
Letrozole
Lomustine
Mechlorethamine hydrochloride
Megestrol acetate
Melphalan hydrochloride
Mercaptopurine
Methotrexate
Methoxsalen
Mitomycin
Mitotane
Mitoxantrone
Nelarabine
Nilotinib
Olaparib
Omacetaxine mepesuccinate
Oxaliplatin

Paclitaxel
Pazopanib hydrochloride
Pemetrexed disodium salt
Pentostatin
Pipobroman
Plerixafor
Plicamycin
Pomalidomide
Ponatinib
Pralatrexate
Procarbazine hydrochloride
Raloxifene
Regorafenib
Romidepsin
Sirolimus
Sorafenib
Streptozocin
Sunitinib
Tamoxifen citrate
Temozolomide
Temsirolimus
Teniposide
Thalidomide
Thioguanine
Thiotepa
Topotecan hydrochloride
Trametinib
Tretinoin
Triethylenemelamine
Uracil mustard
Valrubicin
Vandetanib
Vemurafenib
Vinblastine sulfate
Vincristine sulfate
Vinorelbine tartrate
Vismodegib
Vorinostat
Zoledronic acid

Supplementary Table 2. Information about clinical mTOR inhibitors.*

Compound	Company	CAS No.	Structure	Description	mTORC1	mTORC2	p110α	p110β	p110δ	p110γ	Phase I	Phase II	CML trials	References
**Sirolimus (Rapamycin)**	Pfizer	53123-88-9	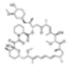	Allosteric	√						12	41	NCT00780104<br>NCT02790515<br>NCT02722668	22
**Everolimus (RAD001)**	Novartis	159351-69-6	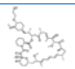	Allosteric	√						15	11	NCT00093639<br>NCT00081874	22
**Temsirolimus**	Wyeth Pharmaceutical	162635-04-3	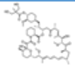	Allosteric	√						4	6		22
**PI-103**	Novartis	371935-74-9	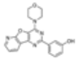	Catalytic	√		√	√	√	√	None†			24
**NVP-BEZ235**	Novartis	915019-65-7	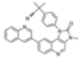	Catalytic	√	√	√	√	√	√	13	1‡	NCT01756118	25
**Gedatolisib**	Pfizer	1197160-78-3	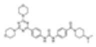	Catalytic	√	√	√			√	3			26, 27
**Apitolisib**	Genetech	1032754-93-0	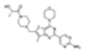	Catalytic	√	√	√	√	√	√	8	4§		28, 29
**VS5584**	Verastem	1246560-33-7	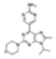	Catalytic	√	√	√	√	√	√	2			30
**AZD8055**	AstraZeneca	1009298-09-2	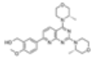	Catalytic	√	√	√	√	√	√	4			31

*Information about ongoing or completed clinical trials were obtained from https://clinicaltrials.gov/.
†PI-103 did not enter clinical trials because of its rapid *in vivo* metabolism (Raynoud *et al*, Cancer Research 2007).
‡1: NCT01658436.
§4: NCT01455493, NCT01442090, NCT01485861, NCT01437566. CAS No; Chemical Abstracts Service (CAS) Number (a unique numerical identifier assigned by CAS).

Supplementary Table 3. Information about clinical samples used in this study.

Patient no.	Additional information
**Pts#1**	No response to imatinib or dasatinib. On ponatinib since 2011 (PACE trial). No CCyR or MMR on ponatinib.
**Pts#2**	Complete hematologic response (CHR) but not CyR on imatinib. Dasatinib started 2007. BCR-ABL 16% in July 2013.
**Pts#3**	Dasatinib ,nilotinib and ponatinib failure. Achieved CHR but no CyR on Bosutinib.
**Pts#4**	TKI failure. Entered accelerated phase with basophilia, thrombocytopenia and Y253H mutation in 2016.

Supplementary Figures

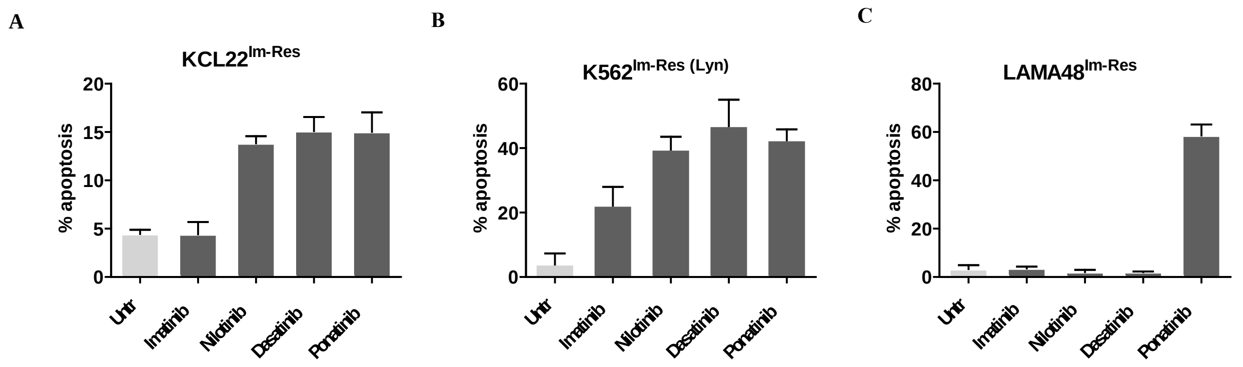

Supplementary Figure 1. Imatinib-resistant CML cells are sensitive to ponatinib. Previously described imatinib-resistant CML cell lines (10, 11) were cultured ± 2µM imatinib, 2µM nilotinib, 150nM dasatinib and 100nM ponatinib. Apoptosis was measured following 72h (A-B) or 24h (C) drug treatment. Error bars=SD.

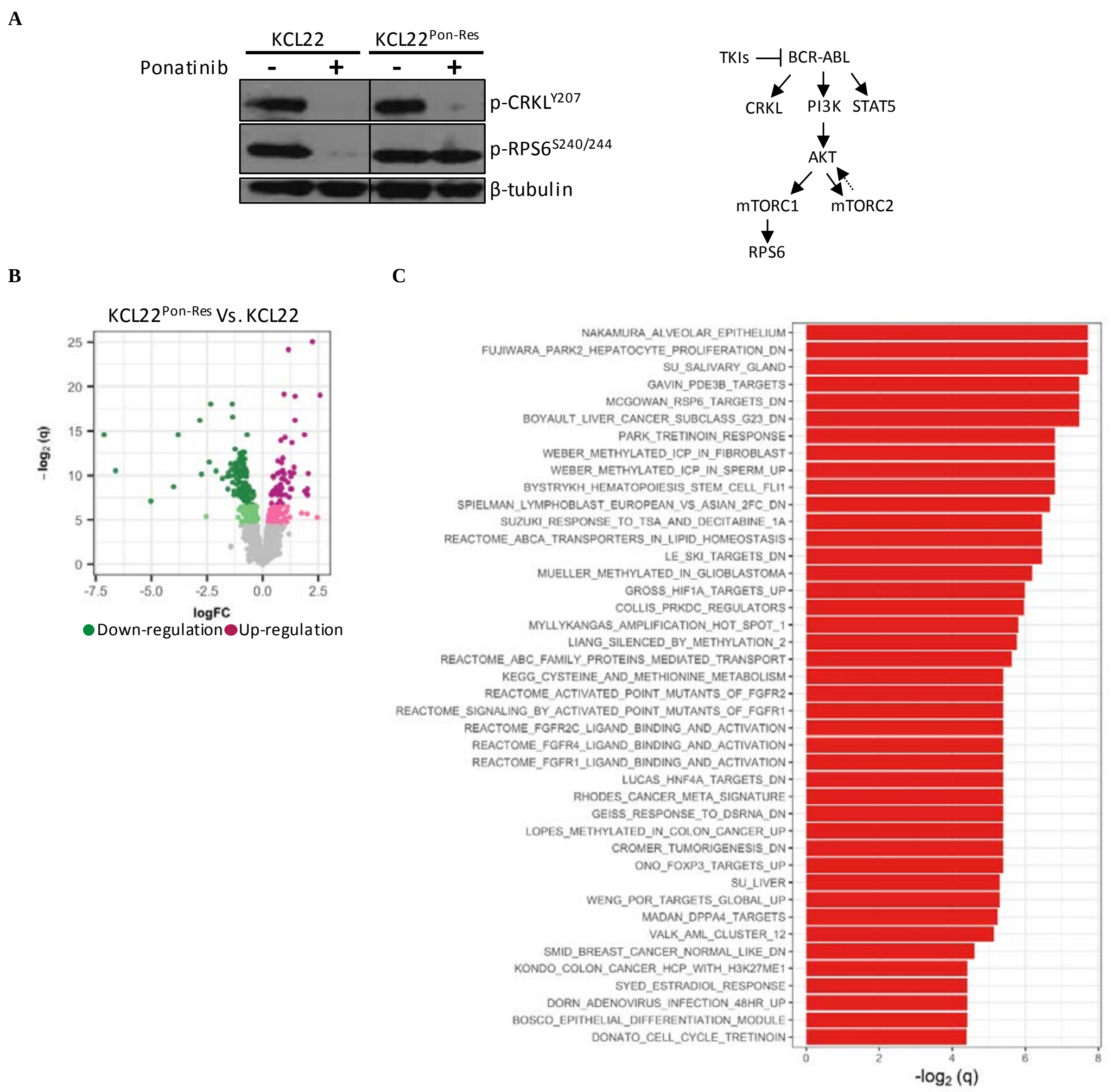

Supplementary Figure 2. Transcriptomic analysis of KCL22$^{Pon\text{-}Res}$ cells. KCL22 and KCL22$^{Pon\text{-}Res}$ cells were left untreated (B-C) or cultured ± 100nM ponatinib (A). Phosphorylation of CRKL and RPS6 was measured after 24h drug treatment (A, left). Schematic diagram demonstrating the BCR-ABL downstream signalling (A, right). The transcriptional difference are represented by Volcano plot (B). Up- and down-regulation transcripts in KCL22$^{Pon\text{-}Res}$ cells are indicated by magenta and green respectively; light and dark colours correspond to q-value thresholds of 0.05 and 0.01 respectively; non-significant changes are coloured grey. Pathways found to be significantly deregulated (q≤0.5) at the transcriptional level in the KCL22$^{Pon\text{-}Res}$ cells when compared with KCL22 cells, ordered by descending q value (C); enrichment is expressed as $-\log_2(q)$.

A

Repurposing Drug	Ponatinib - IC50 (µM)	Ponatinib + IC50 (µM)	
Romidepsin	<0.01	<0.01	
Vincristine sulfate	0.013	0.017	
Ixabepilone	0.014	0.014	
Docetaxel	0.015	0.016	
Cabazitaxel	0.016	0.013	
Vinblastine sulfate	0.016	0.014	
Paclitaxel	0.016	0.014	
Bortezomib	0.019	0.020	
**Omacetaxine mepesuccinate**	**0.042**	**0.023**	
Dactinomycin	0.071	0.073	
Vinorelbine tartrate	0.115	0.370	
**Everolimus**	**0.115**	**0.126**	*
**Sirolimus**	**0.118**	**0.110**	*
Carfilzomib	0.142	0.142	
Clofarabine	0.143	>10	
**Temsirolimus**	**0.158**	**0.189**	*
Plicamycin	0.174	0.174	
Idarubicin hydrochloride	0.217	0.249	
Trametinib	0.287	0.034	
Topotecan hydrochloride	0.345	0.356	
Doxorubicin hydrochloride	0.426	0.793	
Gemcitabine hydrochloride	0.458	>10	
Daunorubicin hydrochloride	0.478	0.602	
Epirubicin hydrochloride	0.498	0.896	
Mitoxantrone	0.529	0.919	
Pralatrexate	0.804	>10	
Thioguanine	0.980	1.339	
Belinostat	1.039	0.840	
Teniposide	1.193	2.511	
Cytarabine hydrochloride	1.964	>10	
Mitomycin	2.468	3.615	
Valrubicin	3.410	5.123	
Crizotinib	4.697	9.238	
Vorinostat	5.170	1.549	
Mercaptopurine	5.203	>10	
Sunitinib	6.521	9.877	
Bleomycin sulfate	6.730	>10	
**Bosutinib**	**8.123**	**>10**	
**Ponatinib**	**>10**	**>10**	
**Nilotinib**	**>10**	**>10**	
**Imatinib**	**>10**	**>10**	
**Dasatinib**	**>10**	**>10**	

B

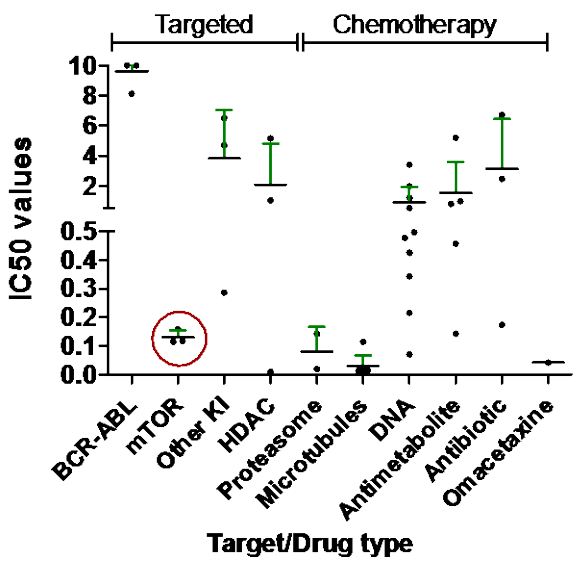

C

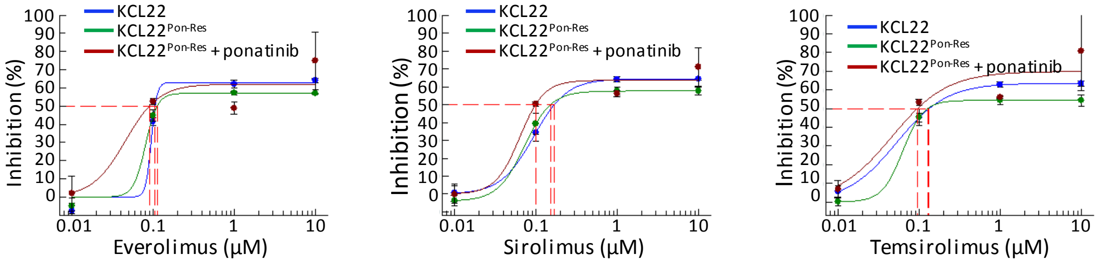

Supplementary Figure 3. Approved oncology drug screen in KCL22$^{Pon\text{-}Res}$ cells. Approved oncology drug library was screened against KCL22 and KCL22$^{Pon\text{-}Res}$ cells (A-C). Drugs were diluted in media and added to cell plates at a final concentration of 0.01, 0.1, 1 and 10µM. Following 72h drug treatment metabolic activity/proliferation was assessed using resazurin

assay and IC50 calculated for each drug used and a comparison made between KCL22$^{Pon\text{-}Res}$ cells cultured in the absence or presence of 100nM ponatinib. Red asterisks mark allosteric mTOR inhibitors (A). Drug-target association analysis of drugs active against KCL22$^{Pon\text{-}Res}$ cells (B). IC50 for allosteric mTORC1 inhibitors is shown (C).

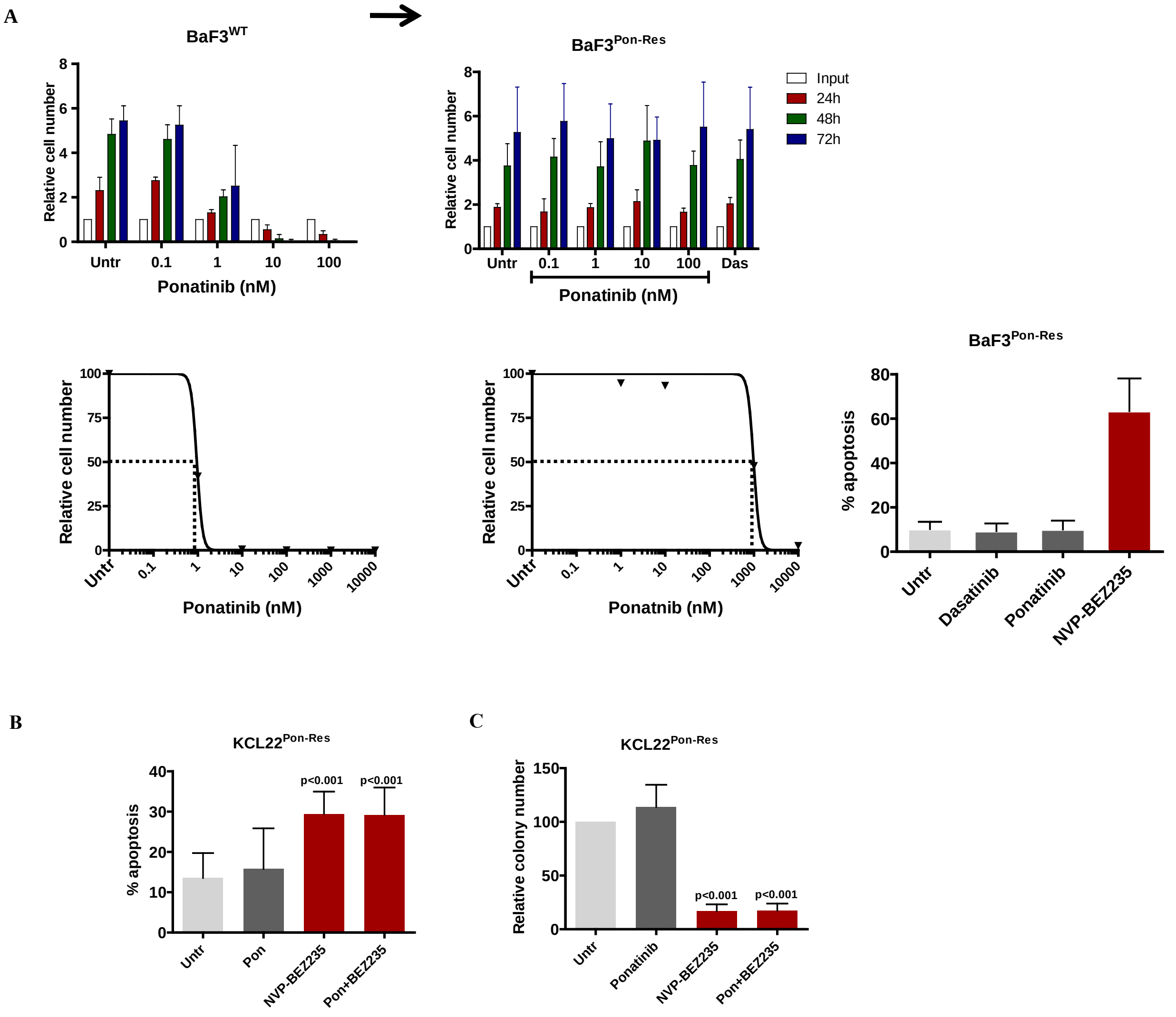

Supplementary Figure 4. Sensitivity of BaF3$^{Pon\text{-}Res}$ cells to NVP-BEZ235. BaF3^{WT} (WT BCR-ABL) and BaF3$^{Pon\text{-}Res}$ cells were cultured ± increasing concentrations of ponatinib and 150nM dasatinib (A). Proliferation was measured following 24, 48 and 72h drug treatment (top panel) and IC_{50} values calculated using GraphPad Prism Software (bottom panel). BaF3$^{Pon\text{-}Res}$ cells were cultured ± 150nM dasatinib, 100nM ponatinib and 100nM NVP-BEZ235 and apoptosis measured following 48h drug treatment (bottom pane, right). KCL22$^{Pon\text{-}Res}$ cells were cultured in 100nM ponatinib alone or in combination with 100nM NVP-BEZ235 (B-C). Apoptosis (B) and colony forming potential (C) were measured following 72h drug treatment. Error bars=SD.

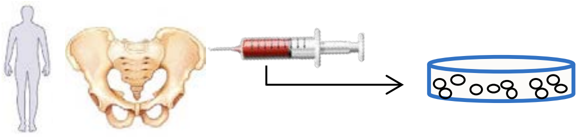

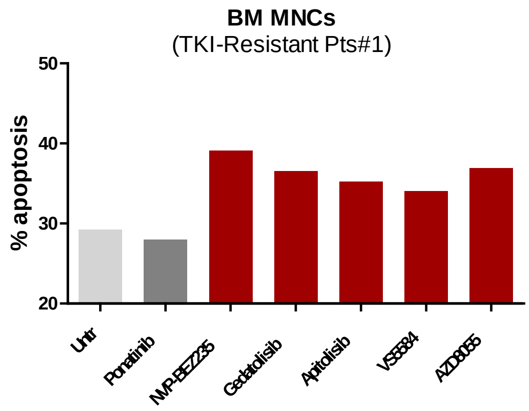

Supplementary Figure 5. Sensitivity of primary MNCs to mTOR inhibitors. BM-derived MNCs from TKI-resistant patient (Pts#1) were cultured in SFM supplemented with PGF and treated with 100nM ponatinib and a panel of catalytic mTOR inhibitors at 100nM concentration (left). Apoptosis was measured following 72h drug treatment (right).

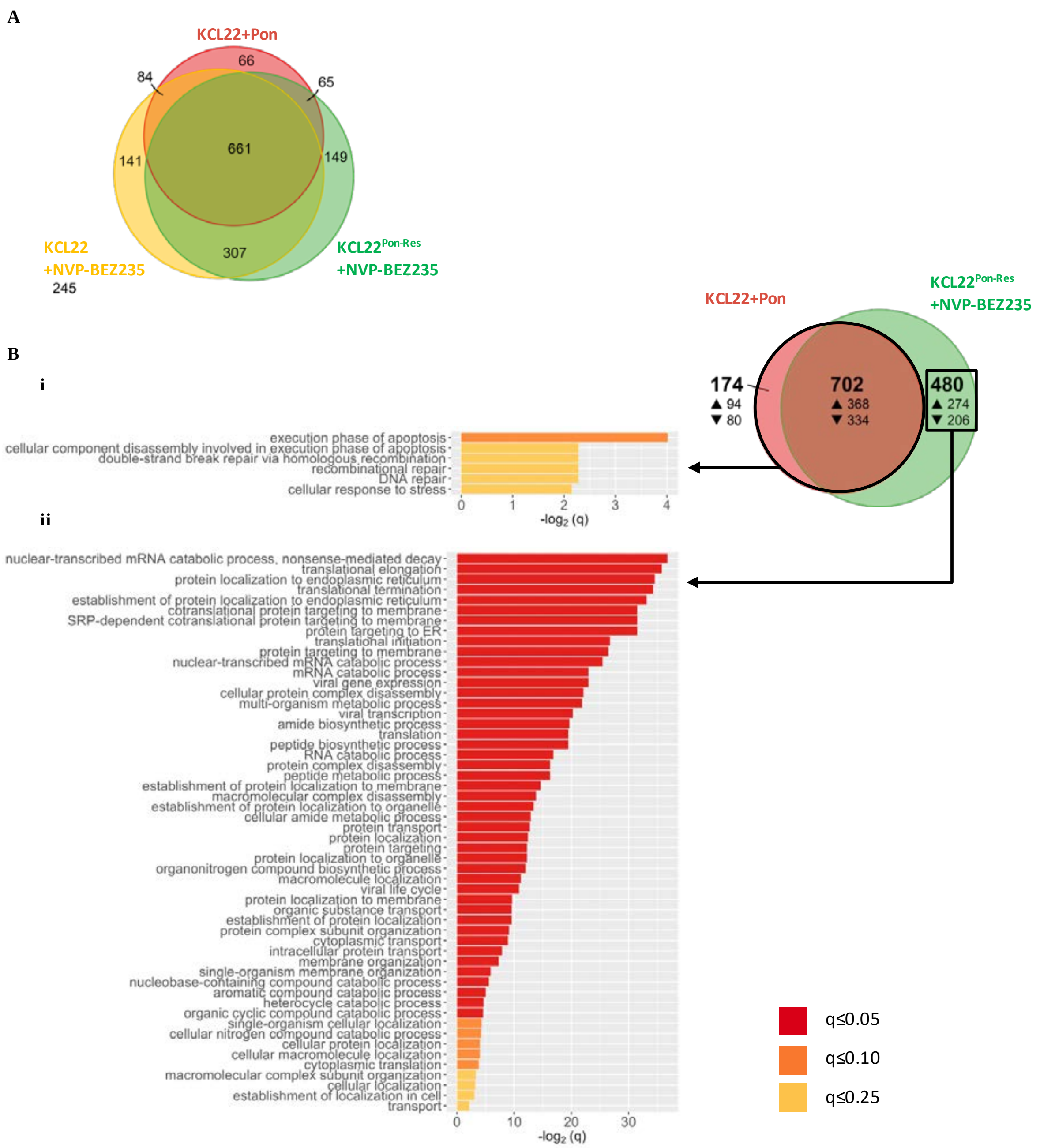

Supplementary Figure 6. Transcriptomic analysis of KCL22$^{Pon\text{-}Res}$ cells. KCL22 and KCL22$^{Pon\text{-}Res}$ cells were cultured ± 100nM ponatinib or 100nM NVP-BEZ235 for 24h and RNA harvested for RNA-seq. The transcriptional response of (i) KCL22 to ponatinib (red); (ii) KCL22 to NVP-BEZ235 (yellow); (iii) KCL22$^{Pon\text{-}Res}$ to NVP-BEZ235 (green) are compared in a proportional Venn diagram (A). Biological Process GO terms found to be significantly overrepresented (q≤0.25) in the transcriptional response of (i) ponatinib in the KCL22 and (ii) NVP-BEZ235 in the KCL22$^{Pon\text{-}Res}$ cells; enrichment is expressed as –log$_2$(q) (B).

A

NVP-BEZ235 (nM)	CQ (μM)	Combined (μM)	Fa	CI
25	2.5	2.525	0.46	1.43124
50	5	5.05	0.57	0.18497
100	10	10.1	0.54	0.59937
150	15	15.15	0.58	0.49557
200	20	20.2	0.61	0.5213

B

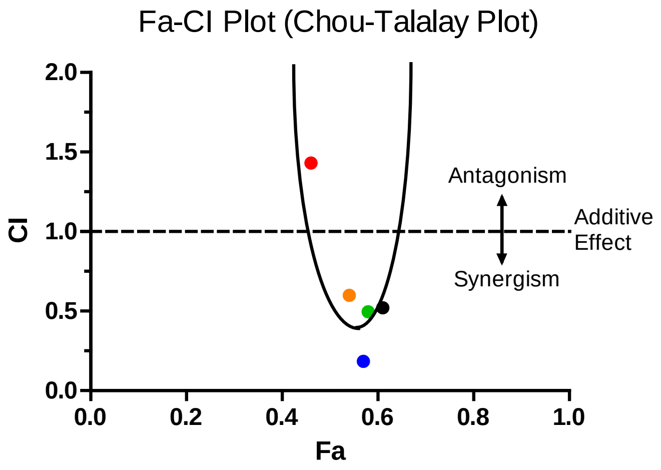

Supplementary Figure 7. Synergism analysis in KCL22$^{Pon\text{-}Res}$ cells. KCL22$^{Pon\text{-}Res}$ cells were cultured with indicated concentration of NVP-BEZ235 and CQ, alone and in combination, for 72h. The fraction affected (Fa) and the combination index (CI) are represented for each drug combination (A). Fa-CI combination index plot for all combination is presented in (B). A combination index of less than 1 indicates synergism; more than 1, antagonism; and 1, additive effect.
